# GI-type T4SS-mediated horizontal transfer of the 89K pathogenicity island in epidemic *Streptococcus suis* serotype 2

**DOI:** 10.1111/j.1365-2958.2011.07553.x

**Published:** 2011-03

**Authors:** Ming Li, Xiaodong Shen, Jinghua Yan, Huiming Han, Beiwen Zheng, Di Liu, Hao Cheng, Yan Zhao, Xiancai Rao, Changjun Wang, Jiaqi Tang, Fuquan Hu, George F Gao

**Affiliations:** 1 CAS Key Laboratory of Pathogenic Microbiology and Immunology, Institute of Microbiology, Chinese Academy of SciencesBeijing 100101, China; 2 Department of MicrobiologyChongqing 400038, China; 3 Department of Biochemistry and Molecular Biology, Third Military Medical UniversityChongqing 400038, China; 4 Graduate University, Chinese Academy of SciencesBeijing 100101, China; 5 Department of Epidemiology, Research Institute for Medicine of Nanjing CommandNanjing 210002, China; 6 Beijing Institutes of Life Science, Chinese Academy of SciencesBeijing 100101, China

## Abstract

Pathogenicity islands (PAIs), a distinct type of genomic island (GI), play important roles in the rapid adaptation and increased virulence of pathogens. 89K is a newly identified PAI in epidemic *Streptococcus suis* isolates that are related to the two recent large-scale outbreaks of human infection in China. However, its mechanism of evolution and contribution to the epidemic spread of *S. suis* 2 remain unknown. In this study, the potential for mobilization of 89K was evaluated, and its putative transfer mechanism was investigated. We report that 89K can spontaneously excise to form an extrachromosomal circular product. The precise excision is mediated by an 89K-borne integrase through site-specific recombination, with help from an excisionase. The 89K excision intermediate acts as a substrate for lateral transfer to non-89K *S. suis* 2 recipients, where it reintegrates site-specifically into the target site. The conjugal transfer of 89K occurred via a GI type IV secretion system (T4SS) encoded in 89K, at a frequency of 10^−6^ transconjugants per donor. This is the first demonstration of horizontal transfer of a Gram-positive PAI mediated by a GI-type T4SS. We propose that these genetic events are important in the emergence, pathogenesis and persistence of epidemic *S. suis* 2 strains.

## Introduction

*Streptococcus suis* serotype 2 (*S. suis* 2) is a Gram-positive, economically important, zoonotic pathogen that is a leading cause of contagious porcine diseases, including meningitis, arthritis, pneumonia, endocarditis and septicaemia (Ma *et al*., 2009; Feng *et al*., 2010). Further, *S. suis* 2 can also be transmitted to humans by direct contact with infected swine, causing meningitis, permanent hearing loss, septic shock and even death (Gottschalk *et al*., 2007). Due to its high incidence, transmissibility, persistence in the environment and its ability to occur in explosive epidemic form, *S. suis* 2 continues to be a global public health concern. After two extensive human outbreaks of streptococcal toxic shock syndrome (STSS) caused by *S. suis* 2 in China in 1998 and 2005 (Tang *et al*., 2006), *S. suis* 2 has been recognized as a re-emerging zoonotic pathogen and was found to characteristically harbour a candidate pathogenicity island (PAI) designated 89K (Chen *et al*., 2007). Recent genetic evidence showing that a two-component system (SalK/R) encoded within this island mediates a piglet-lethal phenotype has strengthened the case that 89K is a PAI (Li *et al*., 2008).

As the name implies, 89K is approximately 89 kb in length, flanked by a perfect 15 bp direct repeat, and contains a putative phage-related integrase gene at its 3′ end. These architectural features strongly suggest that it may have been acquired by horizontal gene transfer (HGT). Meanwhile, the internal modular organization of 89K indicates that it was generated by multiple recombination events because it carries several unrelated gene blocks found in widely divergent species, making it difficult to ascertain its initial origin. A better understanding of the molecular biology of 89K will undoubtedly provide important insight into the emergence, virulence, and evolution of this important pathogen. Indeed, it may reveal some clues concerning the molecular pathogenesis of STSS caused by *S. suis* 2.

It is well recognized that type IV secretion systems (T4SSs) play a key role in HGT, thus contributing to the generation of genomic diversity, the evolution of new pathogenic strains, the dissemination of antibiotic resistance genes and other virulence traits (Christie *et al*., 2005; Juhas *et al*., 2008). Based on the organization of genetic determinants, shared homologies and evolutionary relationships, T4SSs can be classified into three major types: (i) type IVA systems resemble the archetypal VirB/VirD4 system of *Agrobacterium tumefaciens* and are considered to be the paradigm of T4SS; (ii) type IVB systems resemble the Dot/Icm system found in *Legionella pneumophila* and *Coxiella burnetii*; and (iii) other T4SSs bear little or no homology to Type IVA or IVB systems, and their functions await further investigation. Representative of the third group are the recently described genomic island (GI)-type T4SSs that are found in a broad spectrum of Gram-negative pathogens (Juhas *et al*., 2008). Ultimately, it is the identification of the GI-type T4SSs that brings insight into the mechanisms by which GIs transfer between bacteria (Juhas *et al*., 2009).

Here, we present a detailed molecular characterization of the function and evolution of the 89K PAI in epidemic isolates of *S. suis* 2*.* We provide genetic evidence showing that an 89K-borne integrase mediates site-specific excision and circularization of 89K, with contribution from an auxiliary protein named excisionase. The 89K extrachromosomal circular excision product is self-transmissible and can conjugally transfer via a plasmid-like process into *S. suis* recipient cells through the 89K-encoded GI-type T4SS transport conduit. Thus far, while mobilization of genetic elements by T4SSs has been well studied in Gram-negative bacteria, the transfer of Gram-positive mobile genetic elements by T4SSs is currently limited to conjugative plasmids and transposons. Without a previous comprehensive study, we present the first demonstration of the horizontal transfer of a PAI mediated by a GI-type T4SS in a Gram-positive bacterium. We suggest that this new mobile element is responsible, at least in part, for the recent STSS outbreaks in China and recent sporadic cases in neighbouring countries and areas including Vietnam and Thailand (Holden *et al*., 2009), and it will possibly spread further in the future.

## Results

### 89K is transmissible between *S. suis* 2 strains

It is now well established that PAIs comprise a large family of mobile genetic elements and may undergo horizontal transfer to a new host species (Sakellaris *et al*., 2004; Qiu *et al*., 2006; Coburn *et al*., 2007). To determine whether the 89K PAI is transmissible from a donor *S. suis* strain containing 89K to a recipient strain without this element, we performed conjugative mating experiments. To obtain a marked version of 89K, a 3′ single cross-over mutant of strain 05ZYH33 constructed in our previous work was used as the donor, which was tagged with a spectinomycin marker located downstream of the SalK/R two-component system within 89K (Li *et al*., 2008). We designated this derivative 05ZYH33-89KSpc and the modified 89K island as 89K*. SS2-N, a virulent strain of *S. suis* 2 that does not naturally contain 89K, was engineered to be chloramphenicol-resistant by replacing the *recA* gene with a *cat* gene cassette (creating SS2-NΔ*recA*) and used as the recipient. Medium containing both chloramphenicol and spectinomycin was used to select exconjugants that received 89K*. In the filter mating assay, double antibiotic-resistant transconjugants were obtained, and transfer of 89K* was detected at a mean frequency of 5.4 × 10^−6^ per donor (Table 1). Using specific primers, the integrated 89K* was detected in 10 randomly selected transconjugants, providing further evidence that 89K* was transferred into the recipients. Transfer of 89K* was also observed at a similar frequency by using other *S. suis* 2 strains as recipients, including the virulent S735 strain and the avirulent 7996 strain, both isolated from the Netherlands (Table 1). However, conjugation experiments using other serotypes of *S. suis* (serotypes 7 and 9) and other streptococcal species (*S. sanguinis* and *S. agalactiae*) as recipients were performed but without success, suggesting that the 89K transfer may be species- and serotype-specific.

**Table 1:** Conjugal transfer of 89K derivatives between *S. suis* 2 strains

Donor	Recipient	Transfer frequency (per donor)*
05ZYH33-89KSpc	SS2-NΔ*recA*	5.4 × 10^−6^
05ZYH33-89KSpc (DNase I)	SS2-NΔ*recA*	4.9 × 10^−6^
05ZYH33-89KSpc (supernatant)	SS2-NΔ*recA*	< 10^−8^
05ZYH33-89KSpc	S735::*cat*	6.0 × 10^−6^
05ZYH33-89KSpc	7996::*cat*	2.5 × 10^−6^
05ZYH33Δ*mobA89K*	SS2-NΔ*recA*	< 10^−8^
CΔ*mobA89K*	SS2-NΔ*recA*	9.6 × 10^−5^
05ZYH33Δ*mobC89K*	SS2-NΔ*recA*	< 10^−8^
CΔ*mobC89K*	SS2-NΔ*recA*	4.7 × 10^−6^
05ZYH33Δ*virB1-89K*	SS2-NΔ*recA*	9.2 × 10^−7^
CΔ*virB1-89K*	SS2-NΔ*recA*	7.2 × 10^−6^
05ZYH33Δ*virB4-89K*	SS2-NΔ*recA*	< 10^−8^
CΔ*virB4-89K*	SS2-NΔ*recA*	3.8 × 10^−6^
05ZYH33Δ*virB6-89K*	SS2-NΔ*recA*	< 10^−8^
CΔ*virB6-89K*	SS2-NΔ*recA*	4.3 × 10^−6^
05ZYH33Δ*virD4-89K*	SS2-NΔ*recA*	< 10^−8^
CΔ*virD4-89K*	SS2-NΔ*recA*	5.7 × 10^−6^
05ZYH33Δ*int*	SS2-NΔ*recA*	< 10^−8^
CΔ*int*	SS2-NΔ*recA*	4.1 × 10^−6^
05ZYH33Δ*xis*	SS2-NΔ*recA*	8.7 × 10^−8^
CΔ*xis*	SS2-NΔ*recA*	2.9 × 10^−6^
05ZYH33Δ*hlc*	SS2-NΔ*recA*	< 10^−8^
CΔ*hlc*	SS2-NΔ*recA*	6.5 × 10^−6^
05ZYH33 (pVA838-*oriT*)	SS2-NΔ*recA*	6.1 × 10^−4^
Δ*mobA89K* (pVA838-*oriT*)	SS2-NΔ*recA*	< 10^−8^

* These values are the means of three independent experiments.

### Detection of an extrachromosomal circular form of 89K

**Fig. 1:**
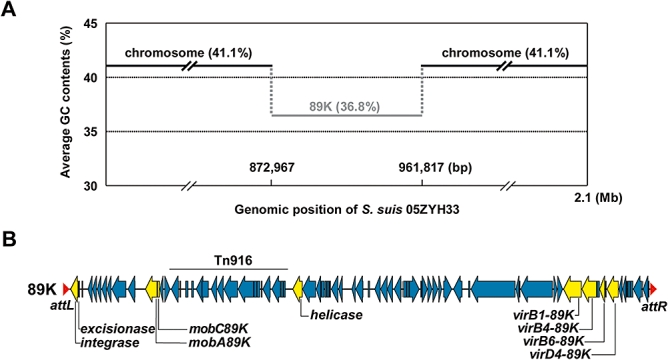
Genetic map of the 89K PAI in the *S. suis* 2 epidemic strain 05ZYH33. A. Aberrant average G+C content of 89K, which is much less than that of the chromosome. B. Gene organization of 89K. The terminal direct repeats *attL* and *attR* are indicated by red triangles. Genes predicted to likely be involved in the mobilization of 89K are shown in yellow. For more gene details, see Chen *et al*. (2007).

To investigate the underlying transfer mechanism, we performed a systematic molecular dissection of the 89K PAI. Based on our previously reported comparative sequence analysis, 89K is located in the genome of *S. suis* strain 05ZYH33 at a position between *05SSU0902* (*hyd*) and *05SSU0983* (*rplL*), which encode a predicted hydrolase and the 50S ribosomal protein L7/L12 respectively (Chen *et al*., 2007). A total of 80 open reading frames (ORFs) were annotated in the 89K island, most of them (*n* = 65) on the minus strand. The overall gene organization in 89K is presented in Fig. 1. Notably, 89K contains a Tn*916* element, which is likely associated with the tetracycline resistance phenotype because the *S. suis* 2 reference P1/7 strain without 89K is sensitive to tetracycline. Furthermore, 89K carries a large lantibiotic biosynthesis cluster, which was suggested to be incapable of producing a functional lantibiotic (due to mutation) but essential for full virulence (Phelps and Neely, 2007; Holden *et al*., 2009).

**Fig. 2:**
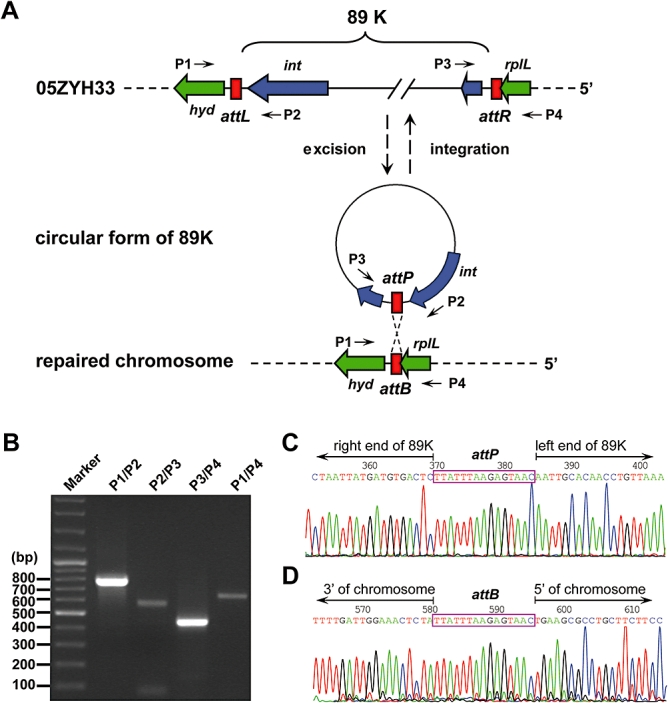
Site-specific integration and excision of 89K in the chromosome of *S. suis* 05ZYH33. A. Schematic representation of the site-specific integration and excision of 89K. The left and right junctions (*attL* and *attR*), which are shown as red rectangles, are formed by recombination between the chromosomal *attB* site and the 89K *attP* site. The flanking genes are shown by solid arrows indicating the direction of transcription. The location and orientation of primers used for detection of integrated and excised 89K are indicated by thin arrows. B. Detection of a circular extrachromosomal form of 89K and the empty chromosomal *attB* site following precise excision, by PCR analysis using primer pairs which are presented upon the lanes, with 05ZYH33 genomic DNA as the template. C. Printout of the sequencing chromatogram of PCR products amplified with primer pair P2/P3, showing the *attP* site (boxed) formed by the joining of the two ends of 89K. D. Printout of the sequencing chromatogram of PCR products amplified with primer pair P1/P4, showing the empty chromosomal *attB* site (boxed) upon 89K excision.

Due to the aberrant GC content of 89K and its absence in the reference P1/7 strain and a number of international virulent strains, it appears that 05ZYH33 acquired the 89K PAI horizontally. 89K then integrated into the chromosome at the *attB* site in the *rplL* gene, generating two direct repeats (*attL* and *attR*) at both ends of the island. It is well documented that integrative and conjugative elements (ICEs) are able to excise from the chromosomes to form circular intermediates prior to conjugative transfer into recipient cells (Burrus *et al*., 2002). We confirmed that 89K did excise from the 05ZYH33 chromosome to produce a circular form and the host genome was repaired upon excision because PCR products were obtained for both primer pairs P2/P3 and P1/P4 (Fig. 2B). Subsequent sequence analysis confirmed the expected structures of *attP* and *attB* generated upon site-specific excision (Fig. 2C and D). Noticeably, the amplicons obtained with primer pairs P2/P3 and P1/P4 showed a substantially weaker signal compared to that with P1/P2 and P3/P4, indicating that 89K excision is a rare event. To measure the rate of 89K excision, we developed a real-time quantitative PCR (qPCR) assay and determined that the frequency of 89K excision from an 05ZYH33 overnight culture was ∼0.032%, a rate similar to that observed for SLG excision from the *Streptomyces lividans 66* chromosome (He *et al*., 2007). These data demonstrated that 89K can spontaneously excise from the chromosome and form a circular excision product. Detection of both integrated and circular 89K suggests that the populations of 05ZYH33 carrying this island are heterogeneous. Most cells carry an integrated island, while others contain an extrachromosomal circular one. Essentially, the same results were obtained for 98HAH12, another highly pathogenic *S. suis* 2 strain, which is predominantly responsible for the earlier 1998 STSS outbreak in China (data not shown).

### The *int* gene is essential for 89K excision

**Fig. 3:**
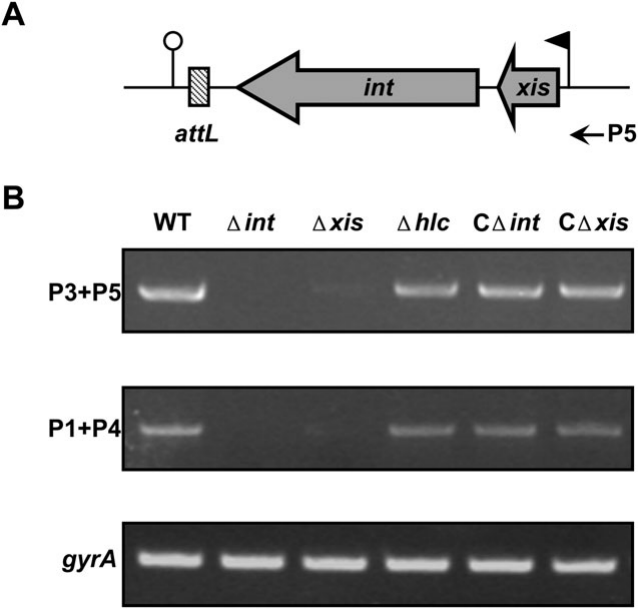
Int and Xis are required for efficient 89K excision. A. Genetic organization of the 3′ terminal region of 89K in *S. suis* 05ZYH33. The *int* gene and the upstream *xis* gene are shown as grey arrows (not drawn to scale). A putative ribosome binding site (RBS) upstream of *xis* is depicted by a small flag. The stem-loop structure represents a putative transcriptional terminator. The position of primer P5 is shown. B. PCR amplicons (indicated left) obtained from the wild-type, mutant and complemented strains (shown above each lane). The *gyrA* gene serves as an internal control.

A common feature of PAIs is the presence of a site-specific recombinase that is presumably responsible for the integration of the PAI into the bacterial chromosome (Mantri and Williams, 2004). The 89K *int* gene, located near the left end of the island (*attL*) at a distance of 63 bp, encodes a 403-amino-acid (aa) integrase belonging to the tyrosine recombinase family, which catalyses site-specific recombination between a chromosomal site (*attB*) and a similar or identical sequence (*attP*) found on mobile genetic elements including bacteriophages, insertion sequences and transposons (Esposito and Scocca, 1997; Nunes-Duby *et al*., 1998). Functional studies of related members of this family give good reason to assume that 89K excision is mediated by the 89K-borne integrase. To determine if this is the case, we generated an isogenic *int* knockout mutant of strain 05ZYH33 (creating 05ZYH33Δ*int*) and examined 89K excision in this mutant. PCR products were generated using primer pairs P1/P5 (primer P5 is located upstream of *xis*, as the *int* deletion removes the priming site for P2) and P3/P4. However, negative results were obtained with primer pairs P3/P5 and P1/P4 (Fig. 3B). qPCR analysis of the Δ*int* mutant also demonstrated that 89K excision was eliminated, with *attP* not being detected. *Trans*-complementation of *int* restored the excision capacity of 89K to a level comparable to that of the parental strain, with *attP* being present in ∼0.025% of cells. These results suggest that deletion of *int* in strain 05ZYH33 abolished the ability of 89K to excise from the host chromosome. Thus, Int is essential for 89K excision and circularization by mediating site-specific recombination between the *attL* and *attR* sites.

### Identification of an auxiliary excision factor, Xis

Based on most of the ICEs that have been examined, the excision function of an integrase is often stimulated by the presence of a closely adjacent recombination directionality factor (RDF), also termed an excisionase (Xis), that both facilitates excision and inhibits integration (Sam *et al*., 2004). In our analysis, a small ORF (*05SSU0904*), located immediately upstream of the *int* gene and separated by a 14 bp intergenic region, was found to encode a possible Xis homologue with 32% aa identity to a putative excisionase (accession number AAL27447.1) from *Enterococcus faecalis* (Fig. 3A). The predicted *xis* is preceded by a putative ribosome binding site (AGGAG), which is located 8 bp upstream from the initiation codon of *xis*. Further sequence analysis revealed a palindrome of 27 bp with the potential to form a stem-loop structure situated 85 bp downstream from the stop codon of *int*, which may function as a transcriptional terminator. The genetic organization of this locus suggests possible translational coupling between *xis* and *int*.

To ascertain whether *xis* is also involved in 89K excision, the excision of 89K was examined in an *xis* deletion mutant of strain 05ZYH33 (named 05ZYH33Δ*xis*). As shown in Fig. 3B, PCR analysis of the excised circular 89K and the repaired chromosome using specific primers both gave faint but still detectable signals in the Δ*xis* mutant, suggesting that there is a very low level of Xis-independent 89K excision. qPCR analysis revealed that *xis* deletion decreased the presence of *attP* to ∼0.0012%, while functional complementation of *xis* restored the excision frequency to ∼0.029%. These results demonstrated that *xis* stimulates, but is not essential for, 89K excision from the chromosome.

### Role of the GI-type T4SS in lateral transfer of 89K

In general, HGT occurs in bacteria through three main mechanisms: transformation, phage-mediated transduction and conjugation (Thomas and Nielsen, 2005). The likelihood of transformation was excluded based on our observation that use of DNase I in the filter mating assay did not abolish the transfer ability of 89K (Table 1). Furthermore, no 89K transfer was detected when the donor culture supernatant was incubated with the recipient, which also ruled out the possibility of phage-mediated transduction (Table 1). Therefore, conjugation is the most likely mechanism for inter-strain transfer of 89K.

Extensive sequence analysis of 89K revealed several ORFs whose putative products show similarity to conjugation and mobilization proteins involved in conjugative DNA transfer (Fig. 1B). On the 3′ half of the 89K island, a peptide encoded by *05SSU0913* showed similarities to various prokaryotic DNA relaxases that initiate conjugal transfer by strand- and site-specific cleavage at the transfer origin (*oriT*), such as the VirD2 component (accession number NP_059814.1) of the *A. tumefaciens* T4SS (36% aa identity) and the MobA relaxase (accession number AAA26445.1) of the broad host-range plasmid R1162 (39% aa identity). Peptide (05SSU0914), located adjacent to this relaxase, shared 36% aa identity to the accessory protein MobC (accession number AAA26444.1) of R1162, which is proposed to act as a molecular wedge, denaturing *oriT* strands and providing a single-stranded (ss) substrate for the relaxase (Zhang and Meyer, 1997). Therefore, *05SSU0913* and *05SSU0914* were accordingly designated *mobA89K* and *mobC89K* respectively.

On the other half of 89K, four gene products display aa similarities to the *Agrobacterium* T4SS components. 05SSU0968 is a VirB1-type lytic transglycosylase (18% identity to the T4SS VirB1 component, accession number NP_059799.1), 05SSU0969 is a putative VirB4-like ATPase (41% identity to the T4SS VirB4 component, accession number NP_059802.1), 05SSU0971 is a hypothetical VirB6-like channel subunit (12% identity to the T4SS VirB6 component, accession number NP_059804.1), and 05SSU0973 is a putative VirD4-like coupling protein (40% identity to the T4SS VirD4 component, accession number NP_059816.1). Thus, *05SSU0968*, *05SSU0969*, *05SSU0971* and *05SSU0973* were accordingly designated *virB1-89K*, *virB4-89K*, *virB6-89K* and *virD4-89K* respectively. Based on these predictions, it was logical to assume that horizontal transfer of the 89K PAI may occur through a GI-type T4SS-mediated conjugation process.

To determine if the 89K PAI harbours a functional GI-type T4SS, mobilization experiments were performed to examine if the transferability of 89K decreased by making individual knockouts of the T4SS gene homologues. Table 1 shows the results of conjugation experiments between these T4SS gene homologue mutants and the recipient strain SS2-NΔ*recA*. No transconjugants were obtained when Δ*mobA89K*, Δ*mobC89K*, Δ*virB4-89K*, Δ*virB6-89K* and Δ*virD4-89K* were used as donors. However, a Δ*virB1*-*89K* derivative of 89K was still able to transfer at a frequency of 9.2 × 10^−7^, approximately sixfold less than that of 89K*. A similar phenomenon had been reported that the transfer capacity of the IncQ plasmid RSF1010 was reduced approximately 10-fold in a *virB1* mutant (Bohne *et al*., 1998). All of these transfer deficiencies could be fully or partially restored by *trans*-complementation (Table 1). Thus, these results indicated that the GI-type T4SS genes are absolutely required for 89K transfer, with the exception of *virB1-89K* (which contributes to but is not essential for 89K transfer).

Parallel conjugative mating experiments were also performed to evaluate if the genes proposed to be involved in the excision and circularization of 89K are also involved in the transfer process. As shown in Table 1, an *int* deletion mutant was transfer-deficient as expected, while the transfer frequency of the Δ*xis* 89K derivative was reduced by almost two orders of magnitude, proximal to the lower limit of detection in our mating system. These observations suggest that the 89K-encoded integrase and excisionase likely cooperatively generate the extrachromosomal circular form of 89K, which represents an intermediate required for the transfer process itself.

### 89K has a functional *oriT* site

**Fig. 4:**
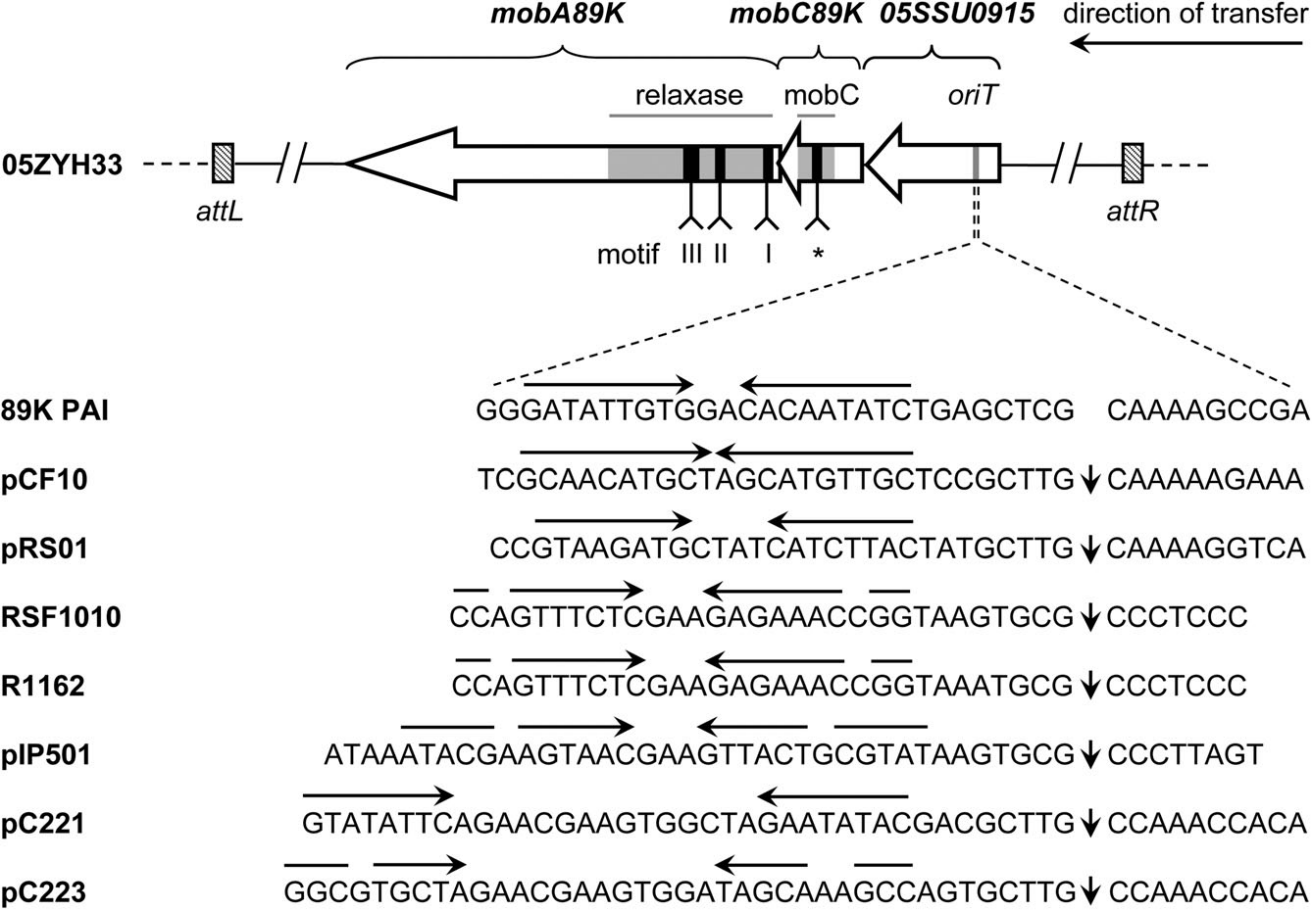
Functional organization of the DNA-processing region of 89K. The mobilization genes (*mobA89K* and *mobC89K*) involved in the DNA processing are marked by open arrows (not drawn to scale). The shaded regions indicate the conserved domains encoded therein, with the internal consensus motifs indicated by black rectangles. The asterisk represents the conserved motif (L/FxxxG/SxNxNQxAxxxN) within the MobC family. The 89K *oriT* was predicted to be positioned within the *05SSU0915* ORF, which encodes a hypothetical protein of unknown function. The direction of transfer is shown by a horizontal arrow. The lower portion of this figure shows the alignment of the 89K *oriT* site with other *oriT* regions of several mobilizable plasmids (indicated left). Arrows above the sequences represent the locations of inverted repeats, and vertical arrows show experimentally determined nick sites.

The transfer of plasmids between bacterial cells by conjugation is the result of two processes: (i) mating-pair formation between the donor and recipient, and (ii) a DNA processing reaction that prepares the plasmid for transfer (Willetts and Wilkins, 1984; Pansegrau and Lanka, 1996). Conjugation can proceed only after the formation of a protein-DNA complex (relaxosome) at the *oriT* site, which is often located in the intergenic region upstream of its cognate relaxase gene (Francia *et al*., 2004). The *oriT* of the 89K PAI was predicted to be a sequence of ∼40 nt positioned within the *05SSU0915* coding sequence, which is predicted to encode a polypeptide of unknown function with 62% aa identity to a hypothetical protein (SMSK597-1197, accession number ZP_07642092.1) of *S. mitis* SK597. This segment contains a perfect 9 nt inverted repeat sequence (predicted by the DNA Strider program) that may be necessary to form a stable nucleoprotein complex during conjugation (Bhattacharjee and Meyer, 1993). The precise *nic* (nickase cleavage) site was predicted to reside at position 3′-(887140)CTCG/CAAA(887147) on the negative strand, which is located 8 nt downstream of the inverted repeats within the *oriT* region based on an extensive sequence alignment with other *oriT* sites that have been experimentally determined previously. Figure 4 shows the overall gene organization of the DNA processing region of 89K. We noticed that MobA89K has an N-terminal relaxase domain but lacks a C-terminal helicase domain, which usually possesses a processive 5′ to 3′ unwinding activity that provides the motive force for strand transfer (Llosa *et al*., 1996; Matson *et al*., 2001). Instead, we found that ORF *05SSU0934* within 89K encodes a match to the DNA helicase (accession number ACF56805.1) of *S. pneumoniae* G54, with 93% aa identity. *In vivo* mutational analysis demonstrated that this gene (designated *hlc*, helicase) is not required for 89K excision and circularization (Fig. 3B) but is essential for 89K transfer (Table 1).

To test the hypothesis that the putative 89K *oriT* sequence plays a crucial role in the initiation of conjugal transfer, a 355 bp segment containing the putative *oriT* site was cloned into a non-transmissible *Escherichia coli*–*S. suis* shuttle plasmid, pVA838. The recombinant plasmid (pVA838-*oriT*) was found to be able to transfer at a frequency of about 6.1 × 10^−4^ transconjugants per donor, which is approximately two orders of magnitude greater than that of 89K*. PCR analysis confirmed the efficient mobilization of pVA838-*oriT* by using specific primers. However, no transconjugants were obtained when the Δ*mobA89K* mutant was used as the carrier donor. These results demonstrated that the non-conjugative plasmid pVA838 could be mobilized only by 05ZYH33 carrying an active relaxase after introducing a fragment encompassing the putative 89K *oriT*. In other words, the 89K PAI has a functional *oriT* site recognized by its cognate relaxase, MobA89K.

### MobA89K and its N-terminal relaxase domain display specific nicking activity in the presence of the MobC89K accessory protein *in vitro*

To gain insight into the relaxation process of 89K, His_6_-tagged MobA89K, MobC89K and the first 258 aa of MobA89K (designated MobA89KN258) were expressed and purified. When overexpressed at low temperature (16°C), MobA89K and MobC89K were both mainly present in the soluble fractions. Although a majority of the MobA89KN258 protein was present in the inclusion body fractions, sufficient soluble material was produced to recover useful amounts of active protein. The molecular masses of the expressed recombinant proteins agreed well with those predicted. All proteins were purified to > 95% homogeneity after metal ion chelating chromatography and gel filtration (Fig. S1).

**Fig. 5:**
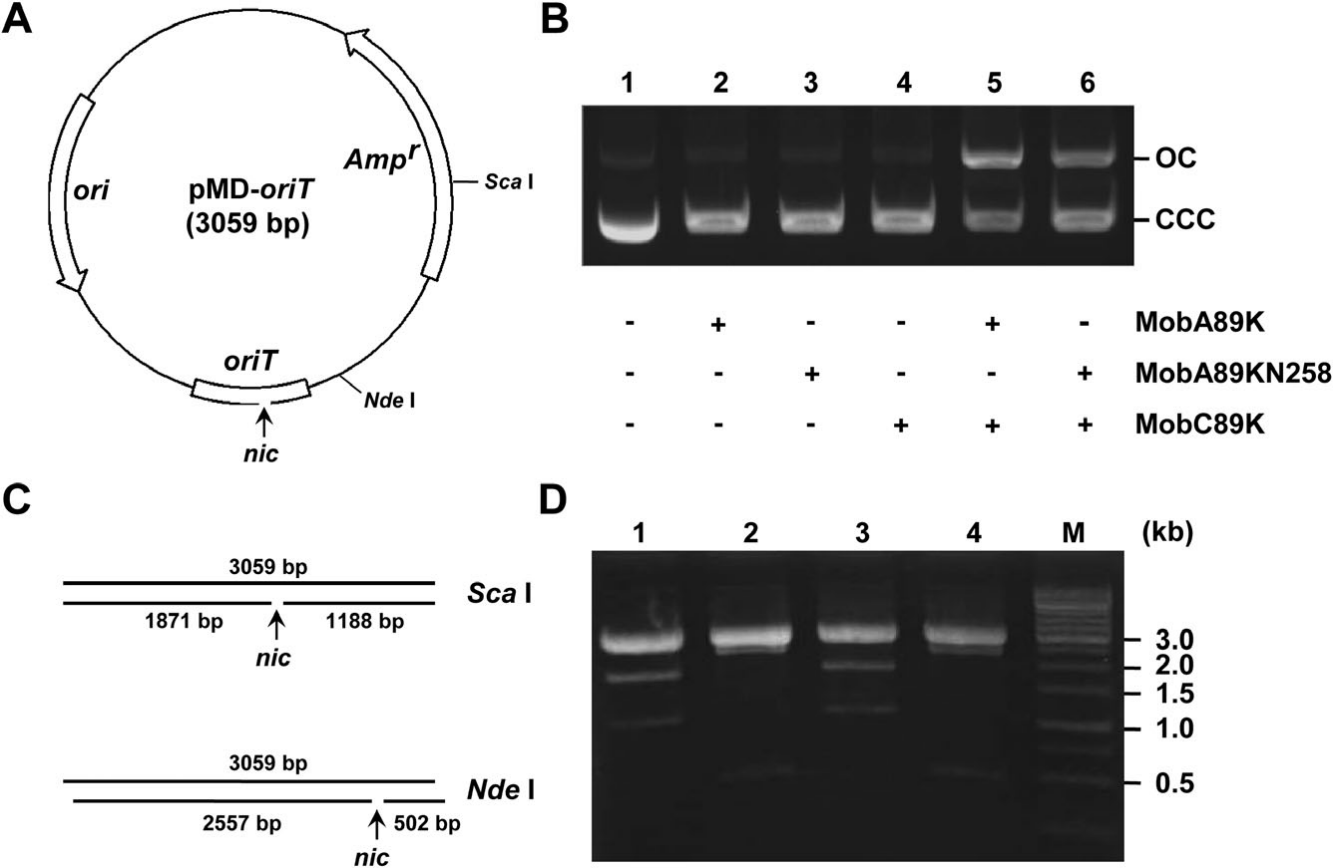
Site- and strand-specific relaxation of the 89K *oriT* region. A. Physical map of the pMD-*oriT* plasmid, which contains the *oriT* region of 89K and serves as substrate DNA in the relaxation analysis. The putative *oriT* nick site (*nic*) is indicated by a vertical arrow. B. Equivalent substrate pMD-*oriT* plasmid was incubated with (+) or without (–) the proteins of interest. Reaction products were analysed by standard agarose gel electrophoresis (0.9%). OC, open circular plasmid DNA; CCC, covalently closed circular plasmid DNA. C. Schematic representation of the expected single-strand species generated by a site-specific nick (arrow) on endonuclease-linearized pMD-*oriT* DNA. The size of each ssDNA fragment is shown. D. pMD-*oriT* was linearized with either ScaI or NdeI, and the relaxation mixtures were analysed on a 0.9% alkaline agarose gel. Lanes: 1, ScaI-linearized pMD-*oriT* with MobA89K and MobC89K; 2, NdeI-linearized pMD-*oriT* with MobA89K and MobC89K; 3, ScaI-linearized pMD-*oriT* with MobA89KN258 and MobC89K; 4, NdeI-linearized pMD-*oriT* with MobA89K and MobC89K. The 1 kb DNA ladder marker is shown to the right (M).

Relaxosomes are presumably composed of supercoiled plasmid DNA with transfer factors complexed at the *oriT* site (Lanka and Wilkins, 1995). To confirm whether the MobA89K and MobC89K proteins can induce the site- and strand-specific DNA cleavage reaction, the relaxation activities of these purified proteins on supercoiled plasmid DNA were tested. The supercoiled *oriT*-containing plasmid pMD-*oriT* was used as substrate DNA in an *in vitro* cleavage reaction. As shown in Fig. 5B, no relaxation was observed in the absence of either protein component. However, MobA89K showed an obvious relaxation activity in the presence of the accessory protein MobC89K, yielding 56% nicked, open circular (OC) plasmid DNA compared with 10% in the untreated control. These findings suggested that both MobA89K and MobC89K are required for the nicking reaction. Meanwhile, MobA89KN258 also increased the conversion of the covalently closed circular (CCC) plasmid DNA into the relaxed form (OC) by the addition of MobC89K, yielding 45% nicked products (i.e. slightly less than that observed for the full-length MobA89K protein).

In another set of experiments, linearized plasmid DNA was subjected to *nic* site analysis using denaturing agarose gels. The *oriT*-containing plasmid pMD-*oriT* was linearized with a restriction endonuclease (ScaI or NdeI) and then co-incubated with the protein components of interest. The predicted *oriT* nick on each of these specifically linearized fragments is shown in Fig. 5C. As expected, three distinct bands corresponding to the predicted sizes (based on nicking at the predicted *oriT* site) were detected on an alkaline agarose gel, in which the dsDNAs were denatured into single strands (Fig. 5D). The C-terminally truncated MobA89K protein showed a comparable nicking activity to that of the full-length protein. In all cases, the largest fragment corresponded to the full-length plasmid ssDNA, and the different sizes of the two additional bands with higher mobility indicated site-specific cleavage of one of the plasmid strands. Indeed, the sum of the sizes of these two fragments equals one full-length linear ssDNA plasmid. These results, together with the supercoiled plasmid DNA cleavage data described above, demonstrated that the *in vitro* nicking reaction was dependent on the presence of both MobA89K and MobC89K, and the MobA89K-dependent *oriT* nicking activity resides within the N-terminal 258-aa relaxase domain of MobA89K.

## Discussion

Integrase-mediated precise excision of mobile elements from the chromosome typically represents the first step in the lateral transfer process. Such excision events usually lead to the formation of circular episomal products that are intermediates in the packaging of some ICEs (e.g. P4) into phage particles (Bowden and Modrich, 1985), or serve as substrates for conjugative transfer of other elements, such as PAPI-1 (Qiu *et al*., 2006) and SXT (Burrus and Waldor, 2003). Sequence analysis revealed that 89K is not similar to an integrated prophage, suggesting that the excised circular 89K may undergo lateral transfer. Confirmation of this hypothesis was obtained by the observation that transfer of a Spc^R^-marked 89K occurred at a frequency of approximately 10^−6^ per donor, similar to that of the *Pseudomonas clc* element (Sentchilo *et al*., 2003) and the *Salmonella* SGI1 island (Doublet *et al*., 2005). Further, 89K transfer absolutely requires the 89K-borne integrase, while a Δ*xis* derivative can still transfer at a low but measurable frequency. This suggests that 89K excision is required for its transfer, and Xis may exert its effect by reducing the number of copies of excised circularized 89K molecules. Notably, Gilmore and co-workers demonstrated that transfer of the enterococcal PAI did not require the PAI-resident integrase, excisionase or putative conjugation genes. Instead, transfer only occurred from donors possessing a pheromone responsive-type of conjugative plasmid via an Hfr-like and RecA-dependent mechanism (Manson *et al*., 2010). Chromosomal transfer mediated by this kind of Hfr-like mechanism was regarded as the major evolutionary force driving the genome dynamics of *S. agalactiae* (Brochet *et al*., 2008)*.*

A combination of bioinformatics and functional analyses revealed a novel member of the GI-type T4SS family that plays a key role in the conjugal transfer of 89K. Throughout the past half century, investigations of T4SSs have largely focused on defining the mechanisms of action of several model systems of Gram-negative bacteria, such as the F (IncF), R388 (IncW), RP4 (IncP) and pKM101 (IncN) plasmid conjugation systems and the *A. tumefaciens* VirB/VirD4 system. However, little is known about the architectures or mechanisms of T4SSs in Gram-positives (Alvarez-Martinez and Christie, 2009). Although T4SS-mediated mobilization of genetic elements has been characterized in some species of Gram-positive bacteria, such elements are currently limited to conjugative plasmids (e.g. *E. faecalis* pCF10, *S. agalactiae* pIP501 and *C. perfringens* pCW3) and transposons (e.g. *E. faecalis* Tn*916*, *B. subtilis* ICE*Bs1* and *S. agalactiae* Tn*GBS2*). Indeed, transfer of Gram-positive GIs (especially PAIs) by T4SSs has not yet been reported. Our findings expand the repertoire of Gram-positive ICEs that can be mobilized by T4SS, and this is the first characterization of horizontal transfer of a Gram-positive PAI by a GI-type T4SS. In contrast, GI-type T4SSs have been identified in various Gram-negative GIs such as pKLC102, PAPI-1, SPI-7 and the *Pseudomonas clc* element, as well as others derived from a wide variety of Gram-negative plant and animal pathogens, including *Erwinia carotovora* (Chen *et al*., 2008), *Xylella fastidiosa* (Zaini *et al*., 2008), *Photorhabdus luminescens* (Held *et al*., 2007) and *Yersinia pseudotuberculosis* (Collyn *et al*., 2006). The complete set of 24 genes thought to encode the putative GI-type T4SS in *H. influenzae* ICE*Hin1056* has been fully or largely identified in the GIs of the above-mentioned Gram-negative pathogens, suggesting that GI-type T4SSs are highly conserved among these species (Juhas *et al*., 2008). Comparative genomic and functional analysis suggested that 89K encodes a GI-type T4SS at its 5′ terminus (*05SSU0961-05SSU0982*), differing in gene content and organization with the Gram-negative counterparts (Fig. S2). This indicates that there may be an evolutionary divergence of GI-type T4SSs between Gram-negative and Gram-positive bacteria, perhaps due to the differences in cell wall structure.

**Fig. 6:**
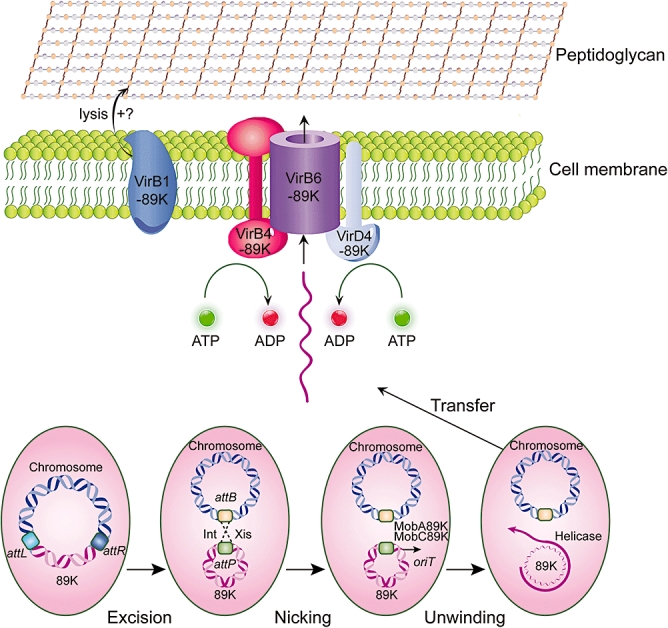
Hypothetical model for the function of the 89K GI-type T4SS. The 89K-borne integrase mediates precise excision of 89K from the chromosome with the aid of excisionase. After receiving a mating signal, the transfer initiates with the *oriT* nicking reaction catalysed by the MobA89K and MobC89K proteins. The cleaved strand is then unwound by the helicase and delivered to the 89K-encoded GI-type T4SS machinery. The VirB1-89K component is thought to act as a lytic transglycosylase, locally opening the peptidoglycan with contributions from some other factor(s) during the assembly of the transport channel. VirD4-89K is a coupling protein required to deliver the DNA substrate to the transport channel, and together with VirB4-89K, required to energize the substrate transport across the channel. The highly hydrophobic VirB6-89K homologue constitutes the main portion of the transport channel.

Based on our experimental results presented here, in combination with previous studies on the *Agrobacterium* oncogenic T-DNA transfer system (Christie and Vogel, 2000; Atmakuri *et al*., 2004; Middleton *et al*., 2005; Guo *et al*., 2007), we would like to propose a model for the transfer mechanism of the 89K PAI (Fig. 6). In a small proportion of host bacteria, the 89K-borne integrase mediates precise excision of 89K from the chromosome with the help of an excisionase. Upon detection of a mating signal, the transfer of the excised circular intermediate of 89K begins (Llosa *et al*., 2002). In the first step, MobA89K initiates the processing reaction by generating a strand- and site-specific nick at the *oriT* site of 89K, with help from the auxiliary MobC89K protein. Next, the 05SSU0934 DNA helicase unwinds the nicked strand during relaxosome formation, and the VirD4-89K coupling protein then links the relaxosome and leads it directly to the mating channel (Llosa *et al*., 2003). The VirB1-89K lytic transglycosylase, for which peptidoglycan cleavage activity has been previously demonstrated in *A. tumefaciens* (Zupan *et al*., 2007), should play a crucial role in local opening of the peptidoglycan during the assembly of the transenvelope transporter complex. However, it should be noted that some other factor(s) (e.g. muramidase) may also be implicated in the formation of the transmembrane pore because inactivation of *virB1-89K* did not completely abolish the conjugal transfer capacity of 89K. VirB6-89K is a highly hydrophobic protein containing six predicted transmembrane segments and is the best candidate for a channel-forming protein in *A. tumefaciens* (Christie, 1997). Adjacent genes encoding peptides (e.g. 05SSU0970, 05SSU0972 and 05SSU0975) harbouring transmembrane domains may also contribute to the construction of the transport apparatus. After the conjugation machinery is manufactured, the VirB4-89K ATPase would work as an alternative translocation energy supplier, aside from VirD4-89K, to motor the DNA substrate through the mating channel and drive export as in *A. tumefaciens* (Dang *et al*., 1999).

Depending on the selective advantages introduced in the recipient variety by the acquired GIs, the elements may undergo insertions, mutations or deletions (Kulasekara *et al*., 2006), and the 89K PAI is no exception. We previously reported that three re-emerging *S. suis* 2 strains in China in 2007 all share the 89K PAI, and two of them had undergone gene losses/deletions (Feng *et al*., 2009). Further, using Roche NimbleGen microarray technology and PCR analysis, we found that several virulent (e.g. 606, 1941 and 8011) and avirulent (e.g. T15 and 2f) *S. suis* 2 strains carry only a portion of this island (unpublished data). The 89K element was also found to be partially included in strain 7996, one of the recipient strains used in our transfer experiments, albeit at a different location. Recently, published work suggests that *S. suis* strain BM407, which was isolated from a human meningitis case in Vietnam in 2004, contains two regions with extended similarity to 89K (Holden *et al*., 2009). Structural and compositional analysis strongly suggests that both of these regions have the potential to undergo excision and transfer, just as 89K does.

Elucidation of the mechanisms involved in bacterial genome instability and, consequently, in the generation of new bacterial variants is still one of the principal goals that have not been completely achieved thus far. Understanding horizontal transfer of GIs is essential to elucidate how genome plasticity in *S. suis* is maintained and how it may contribute to the evolution of infectious diseases. Our work, which demonstrated the horizontal transfer of an important streptococcal PAI, provides an interesting insight into the molecular mechanism of GI transfer and may prove to be exemplary for the study of the evolutionary strategies of other pathogens. A better understanding of the molecular biology of PAIs will both greatly improve our knowledge of bacterial pathogenesis and may lead to new and improved vaccines, novel therapeutics and better environmental monitoring of epidemic strains.

## Experimental procedures

### Bacterial strains, plasmids and culture conditions

The bacterial strains and plasmids used in this study are listed in Table S1. *S. suis* strains were grown in Todd-Hewitt broth (THB) (Difco Laboratories, Detroit, MI, USA) supplemented with 2% yeast extract (THY), and *E. coli* strains were cultured in Luria–Bertani (LB) medium. Agar (1.5%) was included when solid medium was desired. If required, antibiotics were added at the following concentrations: spectinomycin, 100 µg ml^−1^ for both *S. suis* and *E. coli*; chloramphenicol, 5 µg ml^−1^ for *S. suis* and 10 µg ml^−1^ for *E. coli*; erythromycin, 1 µg ml^−1^ for *S. suis* and 250 µg ml^−1^ for *E. coli*; ampicillin, 100 µg ml^−1^ for *E. coli*; kanamycin, 50 µg ml^−1^ for *E. coli*.

### PCR detection of the circular extrachromosomal form of 89K and real-time qPCR assays

To test if 89K can excise from the *S. suis* 05ZYH33 chromosome, a PCR strategy was designed to detect the unoccupied *rplL* locus and an extrachromosomal circular form of 89K using specific primers that face outward from the integrated island or anneal to chromosomal DNA flanking the insertion site (Fig. 2A). Primer combinations P1/P2 and P3/P4 were used to amplify the left and right junction regions respectively. Primer pair P2/P3 was designed to detect the presence of a circular 89K, and primer pair P1/P4 was used to amplify the repaired chromosomal junction formed upon excision. The primers used are listed in Table S2, and PCRs were performed with a standard protocol consisting of 30 cycles. The resulting amplicons were analysed by electrophoresis and were cloned into the pMD18-T vector (TaKaRa) for sequencing.

To determine the frequency of 89K excision, real-time qPCR assays using SYBR Premix Ex Taq (TaKaRa) were developed to measure the percentage of cells in a culture that contained the 89K *attP* sequence. Amplification products were designed to be of similar length to facilitate quantitative comparison. The amount of *attP* DNA in each sample was normalized to that of the chromosomal *hyd* gene located immediately downstream of *attB.* The amplification specificity of each PCR was confirmed by determining melting curves and by electrophoresis of the final PCR products, and efficiency was determined using linearized pMDph plasmid DNA, which contains a single copy of *attP* (157 bp) and an internal region of *hyd* (156 bp) as a template. qPCR reactions and data analysis were performed as described by He *et al*. (2007) and Ramsay *et al*. (2006) respectively.

### Cloning, mutagenesis and genetic complementation

A 355 bp fragment containing the 89K *oriT* sequence was amplified from the 05ZYH33 chromosome using PCR primers oriT-F and oriT-R. The resulting PCR products were cloned into pMD18-T, creating pMD-*oriT*. Plasmid pMD-*oriT* was digested with EcoRI and BamHI to release the *oriT* fragment, which was then cloned into the *E. coli*-*S. suis* pVA838 shuttle vector, generating pVA838-*oriT*. Plasmids pET-MobA89K and pET-MobC89K were created by inserting the *mobA89K* and *mobC89K* PCR products (amplified with specific primer pairs mobA-F/mobA-R and mobC-F/mobC-R respectively) into the NdeI/XhoI sites of the pET-21a(+) expression vector, creating fusion proteins with a C-terminal His_6_ tag. A 774 bp *mobA89KN258* fragment encoding the first 258 aa of MobA89K was also amplified using primers mobA-F/N258-R and cloned into the unique NdeI and XhoI sites of pET-28a(+), creating a fusion protein with His_6_ tags at both its N- and C-termini. All constructs were verified by DNA sequence analysis.

*Streptococcus suis* mutants were generated by allelic replacement with a spectinomycin (*spc*) or chloramphenicol (*cat*) resistance gene cassette, using the method we described previously (Li *et al*., 2008). Genetic complementation was performed by transforming the mutant strain with an *E. coli*–*S. suis* shuttle plasmid (pSET1 or pVA838) construct containing the functional copy of the mutated gene fused in-frame to a cloned *S. suis* suilysin gene promoter (Lun and Willson, 2004).

### Conjugation experiments

Mobilization assays were performed as previously described (Lorenzo-Diaz and Espinosa, 2009a,b;), with minor modifications. Briefly, cultures of donor and recipient cells were grown overnight at 37°C with shaking in 5 ml of THY containing the appropriate antibiotics. Cells were centrifuged and washed to remove the antibiotics, and then diluted 1:10 in pre-warmed fresh THY medium to ensure that cultures were in exponential phase during the matings. Two hundred microlitres of donor culture and 1 ml of recipient culture (1:5 ratio) were mixed in a 1.5 ml Eppendorf tube and spun for 2 min in a microfuge at 14 000 r.p.m. to pellet the cells. The pellet was resuspended in 50 µl pre-warmed THY supplemented with 10 mM MgCl_2_, 2 µg ml^−1^ BSA and 100 U DNase I. The cell mixtures were placed on sterile nitrocellulose filters (0.45 µm, Millipore) and incubated at 37°C for 6 h. Cells were removed by washing the filters in 1 ml of THY medium. Transconjugants were selected on THY medium with spectinomycin and chloramphenicol. Conjugative transfer frequencies were calculated as the number of transconjugant cells per donor. To confirm the occurrence of transfer, 10 transconjugants from each mating were randomly selected for PCR analysis with specific primers.

### Overexpression and purification of recombinant DNA-processing proteins

*Escherichia coli* BL21(DE3) cells harbouring the expression vectors were grown overnight in 50 ml LB broth containing ampicillin or kanamycin at 37°C. A 1:100 dilution of the overnight culture was used to inoculate 2 L fresh LB broth to an OD_600_ of 0.6 and then cooled to 16°C. IPTG was added to a final concentration of 1 mM, and incubations were continued at 16°C for 10 h. Cells were harvested by centrifugation at 6000 *g* for 10 min and resuspended in a lysis buffer containing 20 mM Tris-HCl (pH 8.0), 50 mM NaCl and 5% glycerol. Lysis was completed by sonication on ice, the soluble fractions were collected by centrifugation, and then loaded onto a Ni-NTA affinity column (Qiagen). The proteins were eluted with an increasing concentration gradient of imidazole and then purified by gel filtration chromatography. Peak elution fractions were analysed by electrophoresis on 10% or 12% SDS-PAGE gels. Fractions containing pure proteins were pooled, concentrated in an Amicon apparatus (Millipore) with a 10 kDa molecular weight cut-off membrane, and then stored in 0.1 ml aliquots at −80°C. The concentrations of proteins were determined with a BCA protein assay kit (Pierce) according to the manufacturer's protocol.

### Relaxation assays

In the assay for nicking supercoiled plasmid DNA, reaction mixtures containing 0.2 µmol MobA89K, MobA89KN258 or MobC89K protein, and 300 ng pMD-*oriT* plasmid substrate DNA were incubated in a total volume of 20 µl containing 20 mM Tris-HCl (pH 8.0), 100 mM NaCl, 10 mM MgCl_2_, 0.1 mM EDTA and 2 µg ml^−1^ BSA (Pansegrau and Lanka, 1996). After incubation overnight at 37°C, reactions were stopped by adjusting the mixture to 1% SDS and 0.1 mg ml^−1^ proteinase K, and the incubation was continued at 37°C for 30 min. The reaction mixtures were then subjected to electrophoresis analysis. Gel images were captured digitally, and band intensities were quantified with Quantity One software (Bio-Rad). The yield of nicked OC DNA was calculated after correction for the relative fluorescence of OC to CCC plasmid DNA.

In another set of experiments, linearized plasmid DNA was used as the substrate for the cleavage reaction. pMD-*oriT* plasmid DNA was linearized with an appropriate restriction endonuclease (NdeI or ScaI) that cleaves the plasmid only once before co-incubation with the proteins of interest. The resulting ssDNA fragments were separated on 0.9% alkaline agarose gels, and the amount of cleaved substrate DNA was determined.

### Statistical analyses

Where appropriate, the data were analysed using Student's *t-*test, and a value of *P* < 0.05 was considered significant.
