# Supplementary material for: GI-type T4SS-mediated horizontal transfer of the 89K pathogenicity island in epidemic Streptococcus suis serotype 2
Source: Mol Microbiol. 2011 Mar;79(6):1670–83. doi: 10.1111/j.1365-2958.2011.07553.x (PMC3132442; doi:10.1111/j.1365-2958.2011.07553.x)
Supplement: Supplementary file 1 [file mmi0079-1670-SD1.pdf]

GI-type T4SS mediated horizontal transfer of the 89K pathogenicity island in epidemic *Streptococcus suis* serotype 2

Ming Li[1,2], Xiaodong Shen[3], Jinghua Yan[1], Huiming Han[1,4], Beiwen Zheng[1,4], Di Liu[1], Hao Cheng[1,4], Yan Zhao[2], Xiancai Rao[2], Changjun Wang[5], Jiaqi Tang[5*], Fuquan Hu[2*], George F. Gao[1,4,6*]

[1] *CAS Key Laboratory of Pathogenic Microbiology and Immunology, Institute of Microbiology, Chinese Academy of Sciences, Beijing 100101, China;*

[2] *Department of Microbiology, Third Military Medical University, Chongqing 400038, China;*

[3] *Department of Biochemistry and Molecular Biology, Third Military Medical University, Chongqing 400038, China;*

[4] *Graduate University, Chinese Academy of Sciences, Beijing 100101, China;*

[5] *Department of Epidemiology, Research Institute for Medicine of Nanjing Command, Nanjing 210002, China;*

[6] *Beijing Institutes of Life Science, Chinese Academy of Sciences, Beijing 100101, China.*

*** For correspondence.**

E-mail: gaof@im.ac.cn, Tel: (86-10) 64807688, Fax: (86-10) 64807882 (GFG).

E-mail: hoofuquan@yahoo.com.cn, Tel: (86-23) 68752834, Fax: (86-23) 68752834 (FH).

E-mail: tjq85@hotmail.com, Tel: (86-25) 84526002, Fax: (86-25) 84507094 (JT).

Figure S1. Over-expression and purification of the 89K mobilization proteins. Electrophoresis of the proteins of interest on 10% (MobA89K and MobA89KN258) and 12% (MobC89K) polyacrylamide gels stained with Coomassie brilliant blue R-250. Lanes: 1, non-induced cells; 2, IPTG-induced cells; 3, sonicated supernatant; 4, sonicated sediment; 5, purified protein. Protein size markers are indicated on the left (M). **(A)** MobA89K over-expression and purification. **(B)** MobA89KN258 over-expression and purification. **(C)** MobC89K over-expression and purification.

Figure S2. Comparison of the genetic organization of the GI-type T4SSs present in 89K and several representative Gram-negative GIs with the archetypal *A. tumefaciens* T4SS. GI-type T4SSs were identified in various bacterial species and share only limited homology with the archetypal *A. tumefaciens* T4SS. GI T4SSs are well conserved in Gram-negative-derived GIs. Genes homologous across T4SS groups are shown in the same color. In ICE*Hin1056*, genes involved in pilus formation are shown as gray arrows (not drawn to scale). The gene names in 89K are abbreviated as the last two digits (e.g., gene *05SSU0961 = 61*).

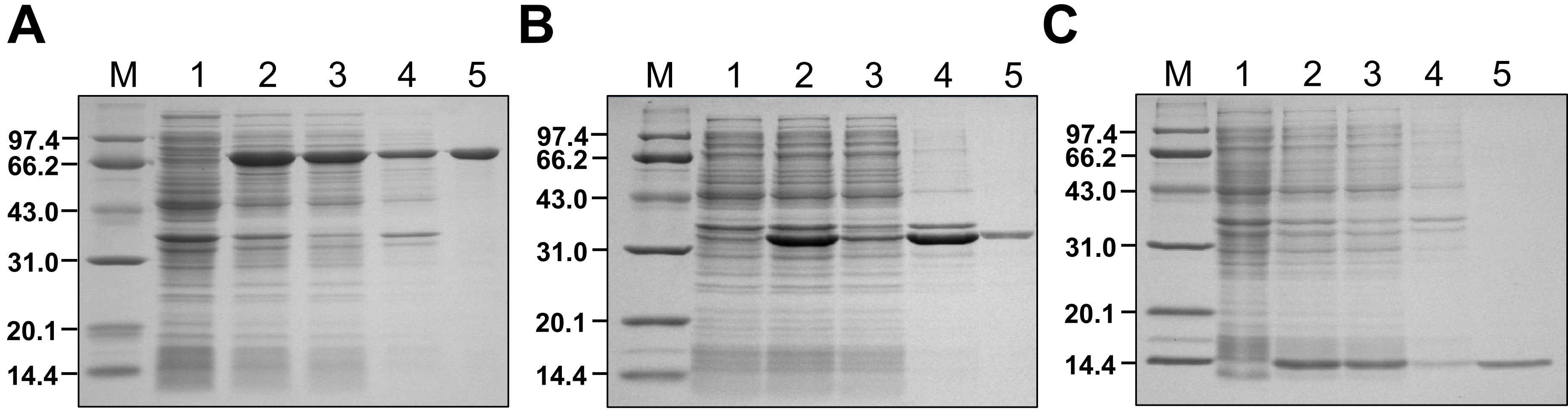
A
M 1 2 3 4 5
97.4
66.2
43.0
31.0
20.1
14.4
B
M 1 2 3 4 5
97.4
66.2
43.0
31.0
20.1
14.4
C
M 1 2 3 4 5
97.4
66.2
43.0
31.0
20.1
14.4

Fig. S1.

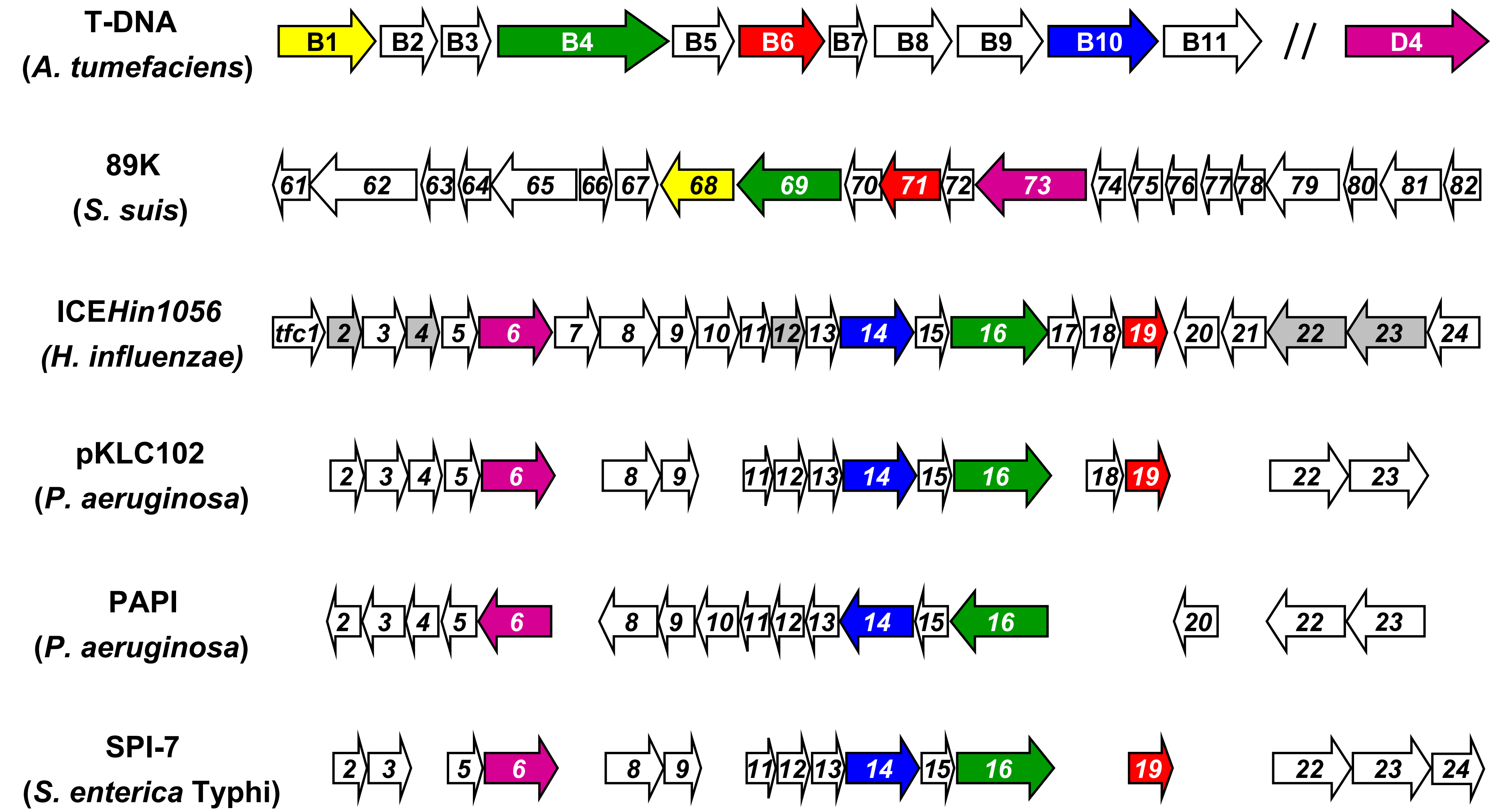
T-DNA
(A. tumefaciens)
B1
B2
B3
B4
B5
B6
B7
B8
B9
B10
B11
D4
89K
(S. suis)
61
62
63
64
65
66
67
68
69
70
71
72
73
74
75
76
77
78
79
80
81
82
ICEHin1056
(H. influenzae)
tfc1
2
3
4
5
6
7
8
9
10
11
12
13
14
15
16
17
18
19
20
21
22
23
24
pKLC102
(P. aeruginosa)
PAPI
(P. aeruginosa)
SPI-7
(S. enterica Typhi)

Fig. S2

Table S1. Bacterial strains and plasmids used in this study.

Strains/plasmids	Characteristics*	Source/reference
**Strains**		
***S. suis* strains**		
05ZYH33	A highly virulent strain isolated from a dead patient with a streptococcal toxic shock-like syndrome	Lab collection
05ZYH33Δ*int*	05ZYH33 derivative with the *int* gene replaced by a *spc* gene cassette; Spc^R	This work
CΔ*int*	Complemented strain of 05ZYH33Δ*int*; Spc^R; Cm^R	This work
05ZYH33Δ*xis*	05ZYH33 derivative with the *xis* gene replaced by a *spc* gene cassette; Spc^R	This work
CΔ*xis*	Complemented strain of 05ZYH33Δ*xis*; Spc^R; Cm^R	This work
05ZYH33Δ*hlc*	05ZYH33 derivative with the *hlc* gene replaced by a *spc* gene cassette	This work
CΔ*hlc*	Complemented strain of 05ZYH33Δ*hlc*; Spc^R; Cm^R	This work
05ZYH33Δ*mobA89K*	05ZYH33 derivative with the *mobA*-like gene *05SSU0913* replaced by a *spc* gene cassette	This work
CΔ*mobA89K*	Complemented strain of 05ZYH33Δ*mobA89K*; Spc^R; Cm^R	This work
05ZYH33Δ*mobC89K*	05ZYH33 derivative with the *mobC*-like gene *05SSU0914* replaced by a *spc* gene cassette	This work
CΔ*mobA89K*	Complemented strain of 05ZYH33Δ*mobC89K*; Spc^R; Cm^R	This work
05ZYH33Δ*virB1-89K*	05ZYH33 derivative with the *virB1*-like gene *05SSU0968* replaced by a *spc* gene cassette	This work
CΔ*virB1-89K*	Complemented strain of 05ZYH33Δ*virB1-89K*; Spc^R; Cm^R	This work
05ZYH33Δ*virB4-89K*	05ZYH33 derivative with the *virB4*-like gene *05SSU0969* replaced by a *spc* gene cassette	This work
CΔ*virB4-89K*	Complemented strain of 05ZYH33Δ*virB4-89K*; Spc^R; Cm^R	This work
05ZYH33Δ*virB6-89K*	05ZYH33 derivative with the *virB6*-like gene *05SSU0971* replaced by a *spc* gene cassette	This work
CΔ*virB6-89K*	Complemented strain of 05ZYH33Δ*virB6-89K*; Spc^R; Cm^R	This work
05ZYH33Δ*virD4-89K*	05ZYH33 derivative with the *virD4*-like gene *05SSU0973* replaced by a *spc* gene cassette	This work
CΔ*virD4-89K*	Complemented strain of 05ZYH33Δ*virD4-89K*; Spc^R; Cm^R	This work
05ZYH33-89KSpc	05ZYH33 derivative with a *spc* gene cassette inserted into the 89K island; Spc^R	(Li *et al.*, 2008)
SS2-N	A virulent strain isolated from a diseased swine in Germany	Lab collection

SS2-NΔ*recA*	SS2-N derivative with the *recA* gene replaced by a *cat* gene cassette; Cm^R,	This work
S735	A virulent strain isolated from a diseased swine in the Netherlands	Lab collection
S735::*cat*	S735 derivative with a *cat* gene cassette inserted into the chromosome	This work
7996	An avirulent strain isolated from a healthy swine in the Netherlands	Lab collection
7996::*cat*	7996 derivative with a *cat* gene cassette inserted into the chromosome	This work
7996::*cat*-89KSpc	A representative transconjugant generated from matings between 05ZYH33-89KSpc and 7996::*cat*	This work
***E. coli* strains**		
DH5α	Cloning host for maintaining recombinant plasmids	Lab collection
BL21(DE3)	Expression host for exogenous protein production	Lab collection
**Plasmids**		
pUC18	Cloning vector; Amp^R	TaKaRa
pMD18-T	T-Cloning vector; Amp^R	TaKaRa
pMDph	Derivative of pMD18-T containing a single copy of *attP* and an internal region of *hyd*	This work
pMD-*oriT*	Derivative of pMD18-T with a cloned 355-bp PCR product containing the *oriT* sequence of 89K	This work
pSET1	*E. coli*-*S. suis* shuttle vector; Cm^R	(Takamatsu *et al.*, 2001)
pVA838	*E. coli*-*S. suis* shuttle vector; Em^R; Cm^R	Lab collection
pVA838-*oriT*	Derivative of pVA838 with a cloned 355-bp PCR product containing the *oriT* sequence of 89K	This work
pET-21a(+)	His-tag fusion expression vector; Amp^R	Novagen
pET-28a(+)	His-tag fusion expression vector; Kan^R	Novagen
pET-MobA89K	Derivative of pET-21a(+) containing the *mobA89K* gene; Amp^R	This work
pET-MobC89K	Derivative of pET-21a(+) containing the *mobC89K* gene; Amp^R	This work
pET-MobA89KN258	Derivative of pET-28a(+) containing a 774-bp fragment encoding the first 258 amino acids of *mobA89K*; Kan^R	This work

* Spc^R, spectinomycin resistant; Cm^R, chloramphenicol resistant; Amp^R, ampicillin resistant; Kan^R, kanamycin resistant.

Table S2. Primers used in this study for PCR and gene expression analyses.

Primers	Sequence (5'-3')	Function
P1	CGCGGAAAATCGTGGCTACT	PCR detection
P2	GTAAAGCGCAGCAAGTAGGAG	PCR detection
P3	AAAATAGCCACGAGATGACACA	PCR detection
P4	TGACATCTGCTGGCGACAAAA	PCR detection
P5	AATGTAATGCGCCAGGAAATG	PCR detection
attP-F	TATCAATCGCTTATCGCTCTCAA	Quantitative PCR analysis
attP-R	GCCAAAATTCCTGCCAAACTT	Quantitative PCR analysis
hyd-F	AGCCGACTGATTTAGAGACTTT	Quantitative PCR analysis
hyd-R	ATTCGTTGAGCATTGGAGAG	Quantitative PCR analysis
oriT-F	GAATTCCGGGGCCACCAAATTATCT (*Eco*R I)	*oriT* clone
oriT-R	GGATCCATGTCTTTTTGGCGGTCTT (*Bam*H I)	*oriT* clone
mobA-F	CATATGGTCATCACTAAGCATTA (*Nde* I)	*mobA89K*/*mobA89KN258* expression
mobA-R	CTCGAGGTATAGTTCCTTATCA (*Xho* I)	*mobA89K* expression
N258-R	CTCGAGTCGGAAATAGCTCTCA (*Xho* I)	*mobA89KN258* expression
mobC-F	CATATGGATTATCGCTACCGCAC (*Nde* I)	*mobC89K* expression
mobC-R	CTCGAGGTGATGACCATAGAAAT (*Xho* I)	*mobC89K* expression
spc-F	GTTCGTGAATACATGTTATA	Amplification of the *spc* gene cassette
spc-R	GTTTTCTAAAATCTGAT	Amplification of the *spc* gene cassette
cat-F	TAATTCGATGGGTTCCGAGG	Amplification of the *cat* gene cassette
cat-R	CACCGAACTAGAGCTTGATG	Amplification of the *cat* gene cassette
